# Promoting Healthy Eating Behaviors by Incentivizing Exploration of Healthy Alternatives

**DOI:** 10.3389/fnut.2021.658793

**Published:** 2021-06-15

**Authors:** Yael Shavit, Yefim Roth, Kinneret Teodorescu

**Affiliations:** ^1^Faculty of Industrial Engineering and Management, Technion—Israel Institute of Technology, Haifa, Israel; ^2^Department of Human Services, University of Haifa, Haifa, Israel

**Keywords:** behavioral change, exploration, diet, eating behavior, healthy lifestyle, salads, decisions from experience, underweighting of rare events

## Abstract

Incentive-based intervention programs aimed at promoting healthy eating behaviors usually focus on incentivizing repeating the desired behavior. Unfortunately, even when effective, these interventions are often short-lived and do not lead to a lasting behavioral change. We present a new type of intervention program focused on incentivizing exploration of new healthy alternatives rather than incentivizing repeated healthy behaviors. This intervention aims to help participants find long-lasting “personal treasures” —new foods that are both healthy and tasty for them. Our field study included a final sample of 48 students with low or medium daily consumption of fresh salads. Participants in the control group received a fixed payment for completing the program, while the participants in the incentivized exploration group received a lower fixed fee for completing the task and a bonus for each new salad they tried. Results show that participants in the incentivized exploration group reported eating more salads even 1 year after the program ended compared to the participants in the control group. Though preliminary, our results paint a promising picture for the successful application of incentivizing exploration interventions to promote healthy lifestyle.

“*It is health that is real wealth and not pieces of gold and silver”*

*Mahatma Gandhi*

## Introduction

It seems that there has never been a better time to maintain a healthy lifestyle. Information is varied and free. If you do not have time to go to the gym, you can work out at home using one of the many free online tutorials. You do not know how to cook? Food will be delivered to you at the click of a button. Yet, although we are surrounded by knowledge about the importance of maintaining a healthy lifestyle, poor diet remains a concern among policymakers. Poor nutrition can be a risk factor for many illnesses such as obesity and obesity-related cancers, type 2 diabetes, cardiovascular disease, osteoarthritis, and depression (1, 2). Therefore, it is of paramount importance to find ways to encourage proper nutrition among the general population.

When trying to promote healthy behaviors, two common intervention approaches are often used: *Communication of knowledge* and *Incentivizing behavioral change*. The communication approach aims to encourage behavioral change by informing and educating participants about the general health benefits of a particular desired behavior. However, such educational approaches, more often than not, lead to long-lasting, enriched, general knowledge that, nevertheless, does not translate into actual behavioral change (3, 4). Incentives-based interventions usually include direct behavior incentivization (e.g., monetary payment), which seems to have some success in promoting healthy behavioral change [e.g., (5)]. However, two meta-analyses regarding weight loss and exercise interventions found no significant effect of monetary incentives after the incentives were removed (6, 7). Thus, both types of interventions appear to be short-lived and do not translate into lifelong changes.

On the one hand, education alone has a hard time changing the incentives to consume healthy foods (e.g., information about potential risks of consuming unhealthy foods does not seem to change behavior) and education is also hard to customize to each individual's subjective preferences. On the other hand, universal external incentives (e.g., monetary) are effective in enhancing healthy behaviors, but once removed; the incentive structure reverts to its original state. Indeed, without direct incentives, the benefit from a healthy option is in the distant future (i.e., better health), while its cost is immediate since healthy foods are commonly considered less tasty (8). Conversely, adopting a healthy lifestyle requires resisting the temptation of the immediate benefit from an unhealthy option (tasty) while emphasizing its cost is in the long-term (health problems). These capabilities/traits might already be weaker in individuals choosing unhealthy lifestyles (9).

Considering this incentive structure, it is perhaps not surprising that the aforementioned intervention schemes have mostly failed. It is well-established that, with respect to intertemporal choices, delayed rewards and costs are weighted as less important (10). Furthermore, a recent study suggests that when intertemporal decisions are made based on repeated experience with an environment, as in most real-life decisions, patience is much reduced (11). Thus, to maintain change over time, one needs to find healthy options that are exceptionally rewarding in several aspects (e.g., tasty, very simple to prepare, cheap, readily available, etc.) to the extent that they are preferred over other more immediately rewarding unhealthy alternatives. Such highly rewarding healthy alternatives (henceforth referred to as Treasures) are particular to each individual (because of subjective individual preferences) and, most likely, not very common.

In the current study, we investigate the problem of establishing long-term healthy lifestyle changes as a problem of under-exploration of rare but highly rewarding and highly individual treasures. A Rare Treasure environment is defined as an environment where the frequent experience following exploration of new options is a little disappointing, but on rare occasions it can lead to the discovery of a highly rewarding Treasure (12, 13). In these cases, people tend to underweight the small probability to find treasures as if they think that rare events are less likely to occur than they really are (14–16). Consequently, people tend to under-explore new alternatives even if exploration is beneficial in the long run (12, 13).

To address the problem of insufficient exploration, we investigate a new type of intervention. Instead of motivating beneficial actions in general, we aim to encourage people to try as many different beneficial options as possible. Enhanced exploration of new beneficial actions increases the chances of finding subjective individual treasures, which equips the person with knowledge about more attractive alternatives to the readily rewarding unhealthy choices. Thus, our experimental intervention draws on the strengths while avoiding the pitfalls of both the common incentives approach (effective but short-lived behavioral change) and the educational approach (long-lasting but subjectively irrelevant knowledge). Incentives have proven effective in short-term behavioral change. Thus, we use monetary incentives to increase exploration of new healthy actions for the duration of the intervention. By not incentivizing repeated actions, we aim to drive subjects to discover their own subjective treasures. Having the subjects self-educate should allow them to gain long-lasting and *subjectively-relevant* knowledge that could translate into actual behavioral change. Importantly, even when the effects of the direct external incentives dwindle, only the incentivized behavior (exploration) is at risk of diminishing along with them, leaving intact the actual healthy behavioral changes that were achieved through the discovery of long-lasting, relevant knowledge.

The potential of incentivizing-exploration interventions is supported by findings from the acquisition of skills literature: One example is the “emphasis change” training protocol (17), according to which the scoring rule is changed on a regular basis, incentivizing trainees to explore new ways to improve performance. This and similar training protocols that encourage exploration were found to enhance performance and transfer of skills among pilots (17, 18), basketball, and hockey players (www.intelligym.com). Similarly, research from the behavioral change literature shows that incentivizing visits to the gym at flexible times led to more gym visits during the intervention period and even after incentives were removed compared to routine incentivizing where participants were rewarded for attending the gym only at a specific time window (19). It seems that exploring different training schedules facilitated participants in finding times that were most convenient for them.

In the current intervention, we aim to promote healthy eating habits by incentivizing exploration of new healthy options. To make the intervention straightforward, we focus only on the consumption of salads containing fresh vegetables which are considered particularly difficult to include in one's diet (20).

## Materials and Methods

This study was approved by an IRB. Approval Number 2017–22.

### Recruitments

We aimed to recruit a relatively homogenous group of people, that do not adhere to a special diet constraint and for whom fresh salads are not an integral part of their daily diet. Thus, we published a short screening questionnaire on Facebook groups of the Technion and the University of Haifa. The questionnaire included several quick questions related to eating habits. One of the questions asked participants about the number of fresh salads they usually eat. After removing those who reported adhering to a specific diet^1^, and those who reported eating more than three fresh salads a week, we had a final inclusion list of 300 students. We invited all 300 students to participate in this study, out of whom 69 responded to the email invitation. Of the 69 students, 55 participants showed up and were included in the final sample.

### Participants

Fifty-five students from the Technion and the University of Haifa participated in the current longitudinal study. Mean age 23.6 (±1.6), 38% females. Two participants were excluded from the experimental group due to a lack of coherence in their responses^2^. Twenty-eight participants were randomly assigned to the control group and 25 to the incentivized exploration group. Five participants did not complete the intervention period leaving us with 22 participants in the incentivized exploration group and 26 participants in the control group.

### Procedure

The intervention lasted 3 weeks. There were three in-person meetings with the participants: pre-intervention (initial meeting), post-intervention (after the 3-week intervention), and a 2-month follow-up. All sessions took place at the Technion or University of Haifa laboratories. Additional follow up 1 year after intervention was conducted via email. Participants received 214 NIS (~$66) on average for participating in the current study. This amount included 30 NIS provided at the end of the pre-intervention meeting, 134 NIS on average that were provided at the end of the post-intervention meeting (depending on the experimental condition, see details below), and 50 NIS for the 2 months follow-up meeting. The 1-year follow-up was incentivized separately (see below).

### Pre-intervention Meeting

Participants signed an informed consent form and then were given a questionnaire (see Supplementary Material 1 for full questionnaire). Among other questions, participants were asked to indicate their fresh salads consumption in the previous week. After filling the questionnaire, the participants were given verbal and written instructions regarding the intervention period according to their experimental group (see Supplementary Material 2). Each participant then received their first payment of 30 NIS.

### The Intervention Period

During the intervention period (3 weeks), participants were asked to eat one fresh salad per day for a minimum of 14 days^3^ and send the experimenter a photo of themselves (“selfie”) with the salad. Participants were also asked to specify the ingredients of the salad and their degree of enjoyment from eating the salad (ranging from “1” not tasty at all to “6” very tasty). Additionally, participants were asked to state whether the salad is entirely new to them (i.e., they did not taste it before or during the study). According to the study definition, a salad is a blend that includes at least 2 freshly cut vegetables and/or freshly cut leaves. Participants were divided randomly into one of two groups with different incentivizing methods.

#### Fixed Payment Group (Control)

Participants in this group received a fixed amount of money (120 NIS/~$37) for completing the requirements during the intervention, meaning eating salads made of at least two fresh vegetables on 14 different days.

#### Incentivized Exploration Group (Experimental)

To encourage exploration of new salads, we manipulated the incentives. Participants were told in advance that their payment will depend on the number of new salads they consume during the study. Each participant received a fixed (65 NIS/~$20) payment for completing the intervention and an additional 7 NIS (~$2) for every new salad they ate. Thus, the more new salads the participants eat, the higher their payment. According to the study definitions, a new salad is a salad for which the ingredients list differs by at least two ingredients from any previously reported salads.

### Post-intervention Meeting

Participants filled a questionnaire (same questionnaire as in the pre-intervention) and answered several questions about the intervention period, such as whether it was easy/difficult for them to find and prepare the salads, how much time per day they spent on the study on average etc. (the questions are available at Supplementary Material 2) Afterward, participants were paid according to the condition they were assigned to and their performance, as explained above.

### Two Months Follow-Up Meetings

Participants filled a questionnaire (same questionnaire as in the pre- and post-intervention) and were then paid 50 NIS.

### One Year Follow-Up

After 1 year, participants completed the final questionnaire (see Supplementary Material 3), which was sent to them online. Out of all participants, five were randomly chosen and paid 100 NIS.

### Statistical Analysis^4^

Statistical analyses were conducted in R 4.0.0 (21) using the *car* (22) and *coin* (23) packages to check test assumptions and the *afex* package for the ANOVA analysis (24). For dependents variables that did not meet the assumption of normality we used non-parametric analysis.

## Results

### Manipulation Check

To examine whether the incentivizing exploration manipulation indeed increased exploration of new fresh salads, we used two types of measures: (1) The number of entirely new salads that participants reported eating during the intervention (one of the questions in the daily reports) and (2) The number of different salads participants ate during the intervention based on the ingredients detailed in their daily reports. Since these dependent variables weren't normally distributed in the collected data, we used the Wilcoxon–Mann–Whitney test.

**Figure 1:**
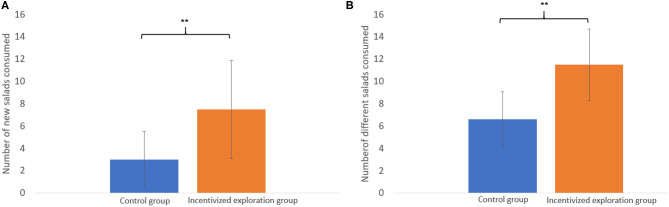
Exploration of salads **(A)** The average number of entirely new salads participants tried during the intervention (based on daily self-reports) and **(B)** the average number of different salads participants ate during the intervention (calculated based on the ingredients detailed in the daily reports). **p < 0.01.

As can be seen in Figure 1A, participants in the incentivized exploration group reported that they tried significantly more entirely new salads (*Med* = 8.5) in comparison to the control group (*Med* = 3, *Z* = 3.18, and *p* < 0.01). Based on the ingredient reports and the study's definition of a different salad (mentioned above), we were able to calculate the number of different salads each participant ate. As can be seen in Figure 1B, this measure also indicated that participants in the exploration group ate significantly more different salads (*Med* = 13) than in the control group (*Med* = 7, *Z* = 4.583, and *p* < 0.001).

### Intervention Effectiveness

To examine whether the incentivized exploration group increased their salad consumption more than the control group, we compared the change in reported salads consumption from the baseline level (pre-intervention) to each of the three-time points (post-intervention, 2 months follow up, and 1 year follow up) between the two groups.

For that aim we conducted a two-way repeated measures ANOVA. The assumption of sphericity was met (assessed by Mauchly's test; *p* > 0.05) and no heterogeneity of variance between the groups was found (assessed by Levene's test; *p* > 0.05). By inspecting the residuals Q–Q plot, it seemed reasonable to assume normality of the residuals.

**Figure 2:**
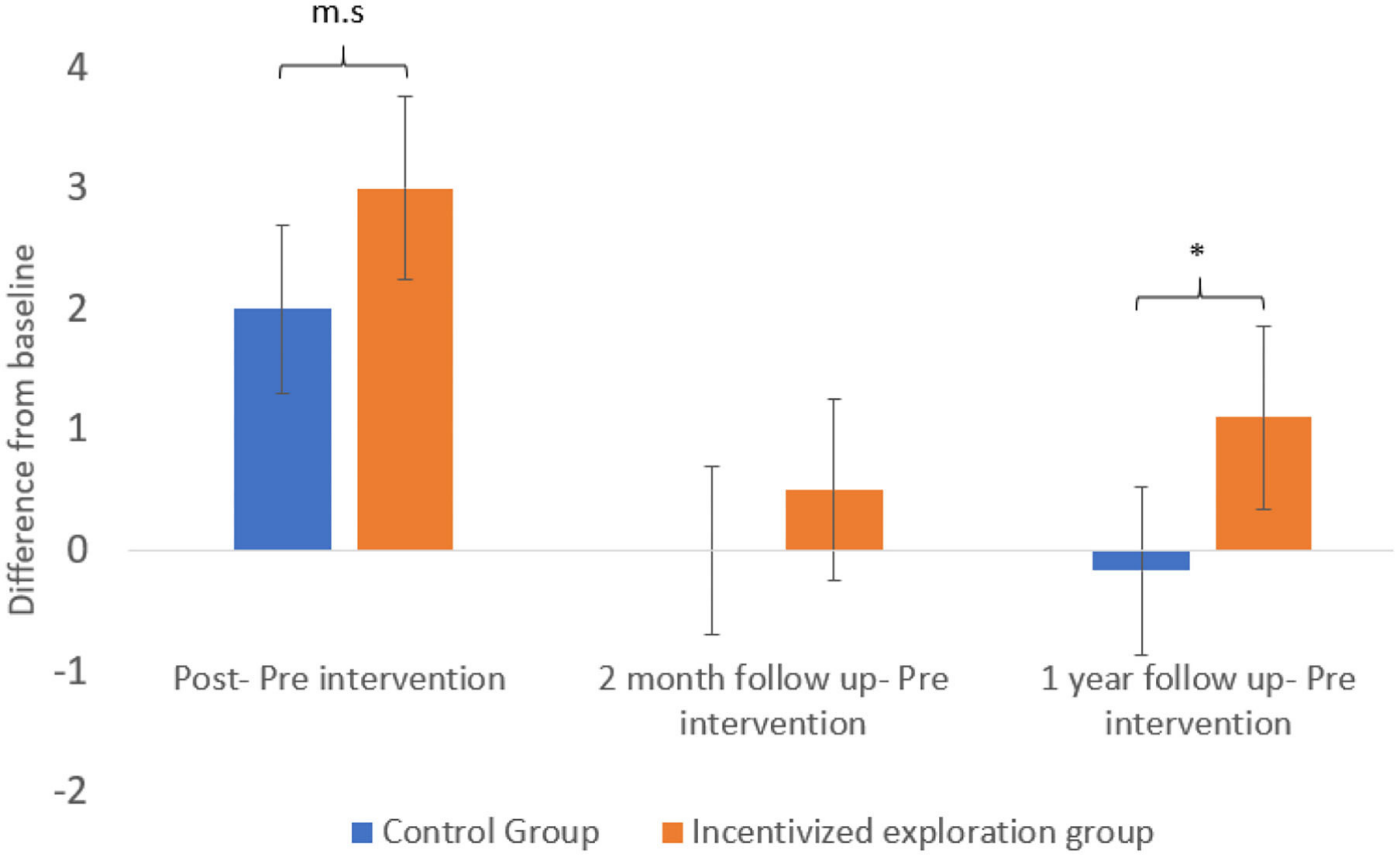
Between groups comparison of the difference between the number of salads eaten pre-intervention and post intervention, 2 months follow up and 1 year follow uptime points. *p < 0.05, 0.051 < m.s <0.1.

Results show a significant effect for group [*F*_(1, 30)_ = 5.2, *p* < 0.05] and for difference in salads consumption [*F*_(2, 60)_ = 36.4, *p* < 0.001]. Simple contrasts analysis revealed that during the intervention (change between pre- to post-intervention reports), participants in the incentivized exploration group increased their salad consumption by three salads on average (SD = ±1.7) compared with two salads on average (SD = ±1.8) in the control group. The difference between the groups was marginally significant [*t*_(30)_ = −1.8, *p* = 0.08]. No significant difference was found between the groups at 2 months follow-up. However, after 1 year, reported salad consumption in the incentivized exploration group increased by 1.1 salads (SD = ±1.3) on average compared with the pre-intervention baseline, while no such increase was observed for participants in the control group. The difference between the groups was significant [*t*_(30)_ = −2.7, *p* < 0.01]. Figure 2 presents the differences between reported salad consumption the week before the intervention and the three-time points.

We conducted a further analysis using repeated measures ANOVA to test for a difference for each group in the number of salads eaten through time. The assumption of sphericity was met (assessed by Mauchly's test; *p* > 0.05) and here too, by inspecting the residuals Q–Q plot, it seemed reasonable to assume normality of the residuals.

**Figure 3:**
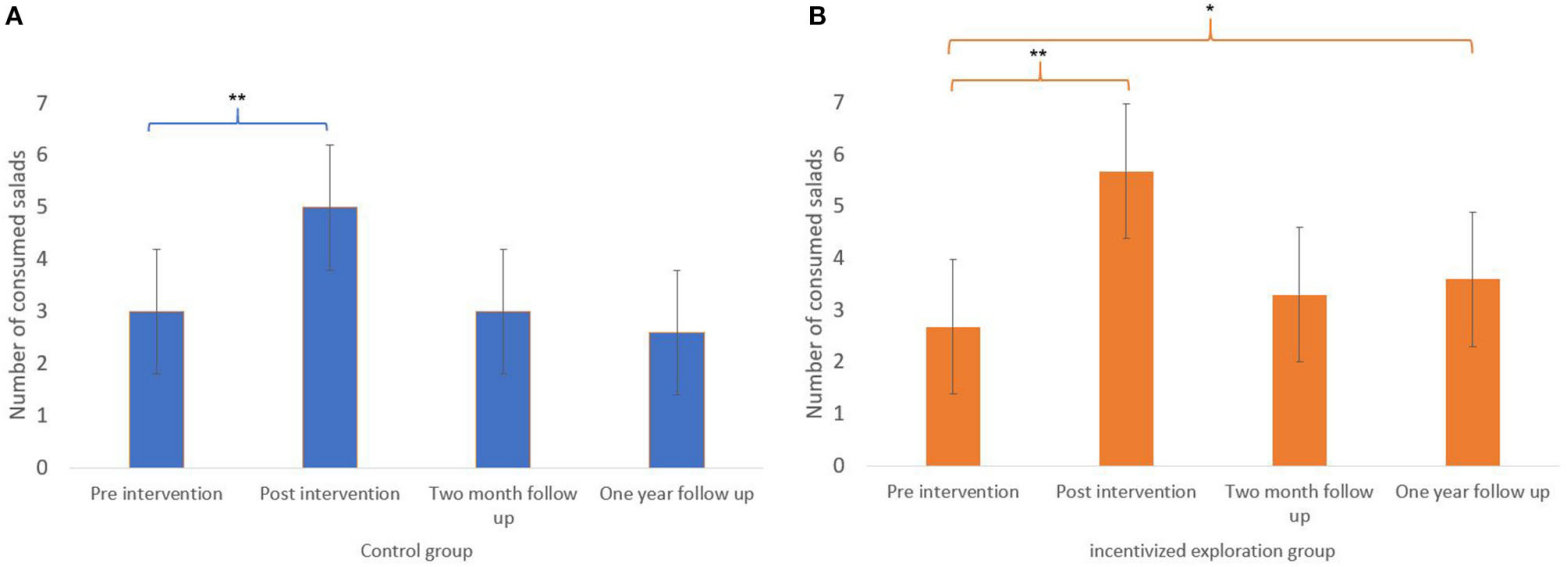
Average amount of consumed salads per week for each group at three time points in comparison to pre intervention self-report. **(A)** Control group. **(B)** Incentivized exploration group. *p < 0.05; **p < 0.001.

Differences were found in both groups [*F*_(3, 48)_ = 13, *p* < 0.001 for the control group and *F*_(3, 42)_ = 38.01, *P* < 0.001 for the incentivized exploration group]. For the control group (as seen in Figure 3A), *post-hoc* tests using the Bonferroni Correction revealed a significant difference in the number of salads eaten the week before pre-and post-intervention meetings, with an average of 2.7 salads (SD ± 0.2) reported in the pre-intervention questionnaire and an average of 5.1 salads (SD ± 0.2) reported in the post-intervention questionnaire (*p* < .001).

As seen in Figure 3B, in the incentivized exploration group, *post-hoc* tests using the Bonferroni correction revealed several significant differences. First, between pre-and post-intervention questionnaires with an average of 2.4 (SD ± 0.3) and 5.9 (SD ± 0.16) salads eaten the week before pre-intervention and the week before post-intervention meeting, respectively (*p* < 0.001). Secondly, a significant difference was also demonstrated between the number of eaten salads the week pre-intervention and the number of salads eaten the week before 1 year follow up meeting (3.6, SD ± 0.036, and *p* < 0.05). There was no difference in the number of salads eaten the week before pre-intervention meeting and the week before 2 months follow up meetings (*p* = 0.26).

### Invested Effort

**Figure 4:**
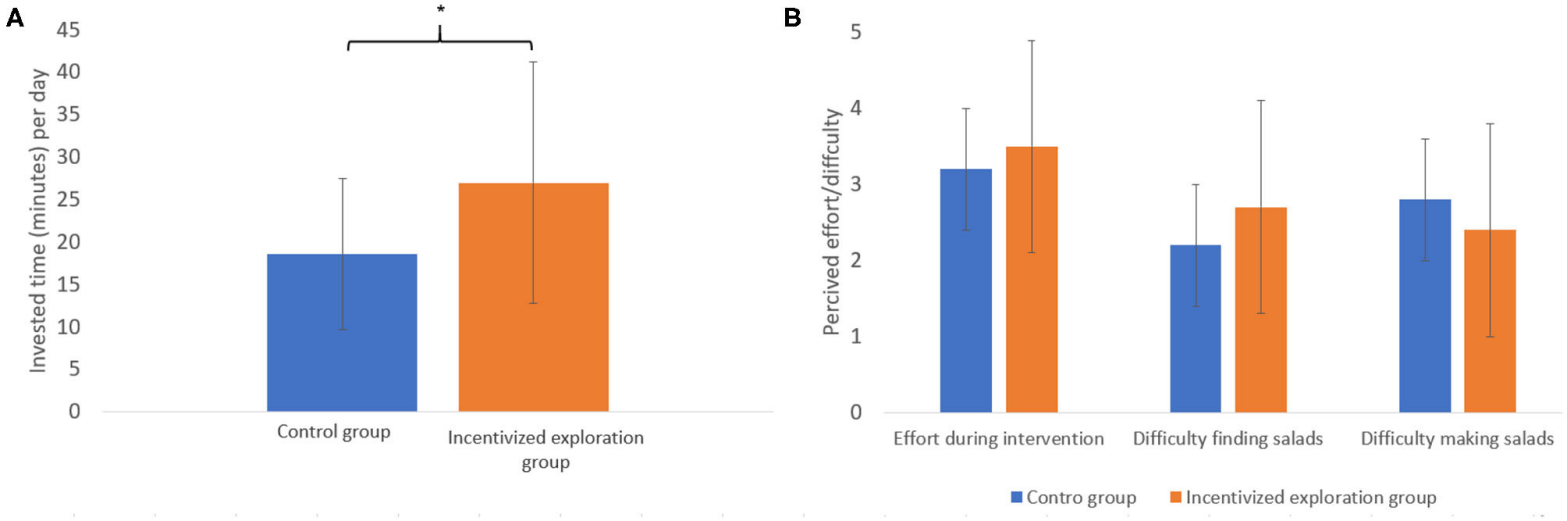
Between groups differences regarding various aspects of effort during the intervention. **(A)** Difference in the average amount of time participants reported spending on the study during the intervention period. **(B)** The average of various aspects of reported self-perceived effort related to the intervention. *p < 0.05.

Figure 4 presents several additional tests aimed at examining the difference between groups regarding various aspects of effort during the intervention. Since these dependent variables weren't normally distributed in the collected data, we used the Wilcoxon–Mann–Whitney test.

Figure 4A presents how much time per day participants reported spending on the study. We found that the incentivized exploration group reported spending significantly more minutes per day (*Med* = 22.5) compared to control group (*Med* = 20, *Z* = 2.4, and *p* < 0.05).

Even so, the three graphs in Figure 4B demonstrate there was no difference in perceived effort during the intervention, in difficulty finding or in making salads (*Z* = 0.6, n.s; *Z* = 1.2, n.s; *Z* = −0.7, n.s, respectively).

## Discussion

Most intervention programs that use incentives to promote desired eating behaviors fail to maintain long-lasting behavioral change (25), possibly because healthy alternatives are difficult to find (26). We argued that incentivizing exploration of new alternatives (in search for rare “Treasures”) instead of indiscriminately incentivizing all desired behaviors increases the chance that people will find highly rewarding and healthy options, thus, helping them maintain healthy eating habits long after incentives were removed.

We compared a common approach of incentivizing behavior to our novel approach of incentivizing exploration. Our results show that participants in the incentivized exploration group, who were incentivized to explore new salads, tried more new salads, and varied their salads during the intervention more than the control fixed-pay group. Most importantly, 1 year after the intervention ended, participants in the incentivized exploration group reported eating more salads than before the intervention, while participants in the control condition did not. These results support our hypotheses that encouraging exploration will [1] lead to finding new rewarding alternatives; and [2] positively affect healthy eating behavior in the long term.

Exploration can be considered risky. Humans tend to fear and reject novel food and consider it dangerous [known as food neophobia (7)]. Indeed, participants in the control group preferred familiar and similar salads. Previous findings suggest that forced exposure to novel food decreased neophobia and increased the willingness to try novel food (27). This emphasizes the importance of incentivizing exploration to encourage search for new foods. It should be noted that exploration is also costly. Participants in the incentivized exploration group spent significantly more time on the intervention. However, the success of interventions encouraging exploration does not require that the exploration of new beneficial actions will continue forever. In fact, most learning models incorporate a transition from initially costly but informative exploration to exploitation of highly rewarding alternatives that were found during exploration (12, 28). In the current context, the aim of the exploration phase is to find a large enough pallet of Treasures one can alternate between. Once this aim is achieved, the new treasures can be exploited and further exploration, although still potentially beneficial, is no longer required to maintain desired behaviors.

Importantly, the cost of exploration could be reflected in various aspects. For example, the effort to find new salads and choose which salad to make, as well as investing more time preparing a wider variety of salads and/or a salad containing a wider range of ingredients. In the present study, the cost of time was measured using only one question that ignores the underlying cause. Future studies may focus on the cost aspect of exploration to examine the relative weights of different cost types.

The main limitation of our study is the small sample size that resulted from the long recruitment process. However, we are encouraged by the fact that we still found significant salads consumption differences, even with our small sample. To the best of our knowledge this is the first evidence for the potential effectiveness of incentivized exploration interventions. Moreover, the effect was observed under minimal intervention, which allowed participants to continue with their everyday routine. Therefore, this type of intervention is relatively convenient, simple, and scalable in duration and intensity, and can be easily adjusted to other types of desired behaviors, such as physical activity.

It is important to note that the current study used monetary incentives to encourage exploration, however, for many people interested in changing the eating habits of others (e.g., nutritionists, parents) paying money is not an option. Nevertheless, our findings relate to the goal that is encouraged and not the medium through which this achieved. Therefore, one may use the tools or methods they normally employ to encourage healthy eating but direct their guidance toward exploration of healthy alternatives rather than just increasing consumption of healthy foods. This may include non-monetary reinforcers such as a good word, screen time, points toward a free consultation etc. The current results suggest that, for example, nutritionists who encourage exploration of alternatives instead of success in general will increase the chances of their patients to find foods that are both tasty and healthy for them. This, in turn, should make these patients more likely to persist in their nutritional change, which is the ultimate goal of nutritional interventions.

## Conclusions

To summarize, the current approach addresses healthy eating as a problem of insufficient exploration for healthy rewarding alternatives. Incentivizing exploration is expected to overcome the problem of insufficient exploration and lead people to discover multiple rare Treasures (heathy and highly rewarding alternatives), which will allow participants to exploit these Treasures long after incentives are removed.

## Data Availability Statement

The raw data supporting the conclusions of this article will be made available by the authors, without undue reservation.

## Ethics Statement

The studies involving human participants were reviewed and approved by Technion IRB Approval Number 2017-22. The patients/participants provided their written informed consent to participate in this study.

## Author Contributions

KT formulated the main idea and provided funding and other resources. KT and YR designed the methodology, reviewed, and edited. YS analyzed the data, wrote the first draft, and collected the data with the help of YR. All authors contributed to the article and approved the submitted version.

## Conflict of Interest

The authors declare that the research was conducted in the absence of any commercial or financial relationships that could be construed as a potential conflict of interest.

## Footnotes

^1^The study took place during the religious holidays Ramadan and Passover. Both involve a specific diet which, can interfere with the intervention. Thus, participants who reported adherence to these specific temporary diets were excluded and were not invited to participate in the current study.

^2^Lack of coherence refers to participants who claimed in their screening questionnaire that fresh salads are not an integral part of their daily diet but in their follow up questionnaire claimed they ate more than three salads a week.

^3^Participants could have eaten as many salads as they wished during the intervention period. The requirement was that they will eat at least one fresh salad on 14 different days (out of the 21 intervention days) and send a photo alongside with the salad ingredients immediately after consuming each of the 14 salads.

^4^Due to the small sample size, subdivision of the groups by gender resulted in even smaller sample size that did not enable us to detect any significant gender differences in our analysis. the possibility of gender differences in behavioral change interventions remains open to future investigations.

## References

[1] Bodnar LM Wisner KL. Nutrition and depression: implications for improving mental health among childbearing-age women. Biol Psychiatry. (2005) 58:679–85. 10.1016/j.biopsych.2005.05.009 16040007 PMC4288963

[2] Dixon JB. The effect of obesity on health outcomes. Mol Cell Endocrinol. (2010) 316:104–8. 10.1016/j.mce.2009.07.008 19628019

[3] Neuhauser L Kreps GL. Rethinking communication in the e-health era. J Health Psychol. (2003) 8:7–23. 10.1177/1359105303008001426 22113897

[4] Glanz K Bishop DB. The role of behavioral science theory in development and implementation of public health interventions. Annu Rev Public Health. (2010) 31:399–418. 10.1146/annurev.publhealth.012809.103604 20070207

[5] Charness G Gneezy U. Incentives to exercise. Econometrica. (2009) 77:909–31. 10.3982/ECTA7416

[6] Paul-Ebhohimhen V Avenell A. Systematic review of the use of financial incentives in treatments for obesity and overweight. Obesity Rev. (2008) 9:355–67. 10.1111/j.1467-789X.2007.00409.x 17956546

[7] Mitchell MS Goodman JM Alter DA John LK Oh PI Pakosh MT. Financial incentives for exercise adherence in adults: systematic review and metaanalysis. Am J Prev Med. (2013) 45:658–67. 10.1016/j.amepre.2013.06.017 24139781

[8] Raghunathan R Naylor RW Hoyer WD. The unhealthy = tasty intuition and its effects on taste inferences, enjoyment, and choice of food products. J Mark. (2006) 70:170–84. 10.1509/jmkg.70.4.170

[9] Ert E Yechiam E Arshavsky O. Smokers' decision making: more than mere risk taking. PLoS One. (2013) 8:1–7. 10.1371/journal.pone.0068064 23844156 PMC3699454

[10] Samuelson PA. A note on measurement of utility. Rev Econ Stud. (1937) 4:155–61. 10.2307/2967612

[11] Shavit Y Roth Y Busemeryer J Teodorescu K. Intertemporal choices in decisions from experience. In: Working Paper Haifa (2021).

[12] Teodorescu K Erev I. On the decision to explore new alternatives: the coexistence of under- and over-exploration. J Behav Decis Making. (2014) 27:109–23. 10.1002/bdm.1785

[13] Teodorescu K Erev I. Learned helplessness and learned prevalence: exploring the causal relations among perceived controllability, reward prevalence, and exploration. Psychol Sci. (2014) 25:1861–9. 10.1177/0956797614543022 25193942

[14] Hertwig R Barron G Weber EU Erev I. Decisions from experience and the effect of rare events in risky choice. Psychol Sci. (2004) 15:534–9. 10.1111/j.0956-7976.2004.00715.x 15270998

[15] Camilleri AR Newell BR. When and why rare events are underweighted: a direct comparison of the sampling, partial feedback, full feedback and description choice paradigms. Psychon Bull Rev. (2011) 18:377–84. 10.3758/s13423-010-0040-2 21327342

[16] Erev I Haruvy E. Learning and the economics of small decisions. Handb Exp Econ. (2013) 2:638–700. 10.1515/9781400883172-011

[17] Gopher D Weil M Siegel D. Practice under changing priorities: an approach to the training of complex skills. Acta Psychol. (1989) 71:147–77. 10.1016/0001-6918(89)90007-3

[18] Seagull FJ Gopher D. Training head movement in visual scanning: an embedded approach to the development of piloting skills with helmet-mounted displays. J Exp Psychol Appl. (1997) 3:163–80. 10.1037/1076-898X.3.3.163

[19] Beshears J Lee HN Milkman KL Mislavsky R Wisdom J. Creating exercise habits using incentives: the trade-off between flexibility and routinization. Manag Sci. (2020). 10.1287/mnsc.2020.3706 PMC8734590 35001975

[20] Ogata K Koyama KI Amitani M Amitani H Asakawa A Inui A. The effectiveness of cognitive behavioral therapy with mindfulness and an Internet intervention for obesity: a case series. Front Nutr. (2018) 27:5–56. 10.3389/fnut.2018.00056 30013974 PMC6036279

[21] R Core Team. R: A Language and Environment for Statistical Computing. Vienna: R Foundation for Statistical Computing (2021). Available online at: http://www.R-project.org (accessed April 29, 2021).

[22] Fox J Weisberg S. An R Companion to Applied Regression, Third Edition. Thousand Oaks CA: Sage (2019). Available online at: https://socialsciences.mcmaster.ca/jfox/Books/Companion/ (accessed April 29, 2021).

[23] Hothorn T Hornik K Van De Wiel MA Zeileis A. A lego system for conditional inference. Am Stat. (2006) 60:257–63. 10.1198/000313006X118430

[24] Singmann H Bolker B Westfall J Aust F Ben-Shachar S. afex: Analysis of Factorial Experiments. R package version 0.28-1 (2021). Available online at: https://CRAN.R-project.org/package=afex (accessed April 29, 2021).

[25] Mantzari E Vogt F Shemilt I Wei Y Higgins JP Marteau TM. Personal financial incentives for changing habitual health-related behaviors: a systematic review and meta-analysis. Prev Med. (2015) 75:75–85. 10.1016/j.ypmed.2015.03.001 25843244 PMC4728181

[26] Roth Y. Do brands serve as reliable signals of nutritional quality? The case of breakfast cereals. J Food Prod Mark. (2017) 23:1–23. 10.1080/10454446.2017.1244787

[27] Pliner P Pelchat M Grabski M. Reduction of neophobia in humans by exposure to novel foods. Appetite. (1993) 20:111–23. 10.1006/appe.1993.1013 8507067

[28] Mehlhorn K Newell BR Todd PM Lee MD Morgan K Braithwaite VA. Unpacking the exploration-exploitation tradeoff: a synthesis of human and animal literatures. Decision. (2015) 2:191–215. 10.1037/dec0000033

