# Supplementary material for: Promoting Healthy Eating Behaviors by Incentivizing Exploration of Healthy Alternatives
Source: Front Nutr. 2021 Jun 15;8:658793. doi: 10.3389/fnut.2021.658793 (PMC8239191; doi:10.3389/fnut.2021.658793)
Supplement: Supplementary file 1 [file Table_1.docx]

Supplemental material 1: Pre- and post- meeting questionnaire^[[1]](#footnote-1)^

Dear Participant

You will now be asked to answer several questions. There are no right or wrong answers. The data collected will only be used for the current study and all your information will remain private.

Demographic information:

1. Participant ID__________
2. First Name____**______**
3. Last name____**______**
4. Age____**______**
5. Degree:

- Undergraduate,
- Graduate,
- Phd

1. Faculty____**______**
2. Marital status:
3. Single
4. In a relationship (living separately)
5. In a relationship (living together)
6. Married
7. Divorced
8. Native language:
9. Hebrew
10. Arabic
11. Russian
12. English
13. French
14. Other ____**______**
15. How much money do you have for personal usage a month (Including salary and parents assistance) ____**______**

(10) What is your height ____**______**

(11) What is your weight ____**______**

(12) How often do you practice aerobics exercise such as running, swimming or bike riding?

1. Less than once a month
2. 1-3 times a month
3. Once a week
4. 2-3 times a week
5. More than 3 times a week

(13) How much time (in minute) does your workout last ____**______**

(14) How often do you practice anaerobic exercise such as weightlifting, yoga, Pilates?

1. Less than once a month
2. 1-3 times a month
3. Once a week
4. 2-3 times a week
5. More than 3 times a week

(15) How much time (in minutes) does your workout last ____**______**

(16) How often do you smoke?

1. I have never smoked
2. I don’t smoke now, but I used to
3. I rarely smoke (for example, only at parties)
4. I smoke regularly but not a lot (less than a pack a week)
5. I smoke less than a pack of cigarettes a day
6. I smoke a pack of cigarettes a day or more

(17) On average, how many hours do you spend in front of a screen (e.g computer, TV, smartphone)?

(1) Less than an hour

(2) Between one and two hours a day

(3) 3-5 hours a day

(4) 5-10 hours a day

(5) 10-15 hours a day

(18) How many hours do you spend in front of a screen consuming entertainment content (for example, surfing Facebook, YouTube, TV, etc.). This question refers to all screen hours that are not related to work / study tasks.

(1) Less than an hour

(2) Between one and two hours a day

(3) 3-5 hours a day

(4) 5-10 hours a day

(5) 10-15 hours a day

(19) How many hours on average do you sleep at night? ____**______**

(20) Lifestyle and nutrition- average:

|  | (1)  Very unhealthy | (2) | (3) | (4) | (5) | (6) | (7)  Very healthy |
| --- | --- | --- | --- | --- | --- | --- | --- |
| How would you define your lifestyle? |  |  |  |  |  |  |  |
| How would you define your diet? |  |  |  |  |  |  |  |

(21) Do you suffer from health problems that cause you to pay special attention to your diet (e.g. allergies, high cholesterol, diabetes)?

1. No
2. Yes, please elaborate

(22) Do you adhere to any dietary rules (for example, diet)?

1. No,
2. Not right now, but in the past
3. Yes

(23) How many times on average, do you eat (drink):

|  | (1) Less than once a week | (2) Once a week | (3) Two times a week | (4) Three times a week | (5) 4-5 times a week | (6) Once a day | (7) 2-3 times a day | (8) More than 3 times a day |
| --- | --- | --- | --- | --- | --- | --- | --- | --- |
| “Junk food" (such as pizza, hamburger, shawarma) |  |  |  |  |  |  |  |  |
| Sweets and snacks (such as cakes, cookies, pastries) |  |  |  |  |  |  |  |  |
| Healthy snacks (such as oatmeal, unsweetened nuts) |  |  |  |  |  |  |  |  |
| Fresh fruits (such as apples, bananas, oranges) |  |  |  |  |  |  |  |  |
| Fresh vegetables not as part of a salad (such as pepper, cucumber, tomato) |  |  |  |  |  |  |  |  |
| Salads (a mixture that includes at least two fresh vegetables and / or fresh leaves) |  |  |  |  |  |  |  |  |
| Alcohol |  |  |  |  |  |  |  |  |

(24) How many different types of food you eat (drink) in each of the following categories during the week:

|  | Do not eat (drink) at all | 1 type | 2 types | 3 types | 4 types | 5 types | 6 types | 7 types or more |
| --- | --- | --- | --- | --- | --- | --- | --- | --- |
| Junk food |  |  |  |  |  |  |  |  |
| Sweets and snacks |  |  |  |  |  |  |  |  |
| Healthy snacks |  |  |  |  |  |  |  |  |
| Fresh fruits |  |  |  |  |  |  |  |  |
| Fresh vegetables not as part of a salad |  |  |  |  |  |  |  |  |
| Salads (a mixture that includes at least two fresh vegetables and / or fresh leaves) |  |  |  |  |  |  |  |  |
| Alcohol |  |  |  |  |  |  |  |  |

(25) How often do you eat out?

1. Less than once a month
2. 1-3 times a month
3. Once a week
4. Twice or three times a week
5. More than three times a week

(26) Do you like to eat (drink):

|  | 1  Very dislike | 2 | 3 | 4 | 5 | 6 | 7  Like very much |
| --- | --- | --- | --- | --- | --- | --- | --- |
| Junk food |  |  |  |  |  |  |  |
| Sweets and snacks |  |  |  |  |  |  |  |
| Healthy snacks |  |  |  |  |  |  |  |
| Fresh fruits |  |  |  |  |  |  |  |
| Fresh vegetables not as part of a salad |  |  |  |  |  |  |  |
| Salads (a mixture that includes at least two fresh vegetables and / or fresh leaves) |  |  |  |  |  |  |  |
| Alcohol |  |  |  |  |  |  |  |

(27) How many meals a day do you usually eat (not including snacks between meals)?

1. One
2. Two
3. Three
4. Four
5. Five or more

(28) Each item in this questionnaire includes a sentence that describes a belief about your health, a sentence with which you can agree or disagree. Next to each sentence, there is a rating ranging from (1) strongly disagree to (6) strongly agree. This questionnaire is a measure of your personal beliefs and therefore, there are no right or wrong answers.

|  | 1. Strongly disagree | (2) | (3) | (4) | (5) | 1. Strongly agree |
| --- | --- | --- | --- | --- | --- | --- |
| If I get sick, it is my behavior that will determine how quickly I will recover |  |  |  |  |  |  |
| No matter what I do, if I'm going to be sick – I will be sick |  |  |  |  |  |  |
| The best way for me to avoid illness is to be in touch with my doctor regularly |  |  |  |  |  |  |
| Most things that affect my health happen to me by chance |  |  |  |  |  |  |
| When I am not feeling well, I should consult a medical professional |  |  |  |  |  |  |
| I am in control of my health |  |  |  |  |  |  |
| My family has a lot of influence on me being sick or healthy |  |  |  |  |  |  |
| When I get sick, I am the one to blame |  |  |  |  |  |  |
| Luck plays a significant role in determining how quickly I will recover from an illness |  |  |  |  |  |  |
| Health professionals control my health |  |  |  |  |  |  |
| My good health is mostly a matter of luck |  |  |  |  |  |  |
| The main things that affects my health are my actions |  |  |  |  |  |  |
| If I take care of myself, I can avoid getting sick |  |  |  |  |  |  |
| When I recover from an illness, it is usually because other people (e.g doctors, nurses, family and friends) have treated me well |  |  |  |  |  |  |
| No matter what I do, I will probably get sick |  |  |  |  |  |  |
| I will stay healthy if it's meant to be |  |  |  |  |  |  |
| If I take the right actions, I can stay healthy |  |  |  |  |  |  |
| Regarding my health, all I can do is what my doctor tells me to do |  |  |  |  |  |  |

(29) For each item below, choose the number that best represents your degree of agreement or disagreement as to whether the sentence is right or wrong for you. This questionnaire is a measure of your personal preferences, and therefore, there are no right or wrong answers.

|  | (1) Strongly disagree | (2) | (3) | (4) | (5) | (6) Strongly agree |
| --- | --- | --- | --- | --- | --- | --- |
| I regularly try new and varied foods |  |  |  |  |  |  |
| I do not trust new foods |  |  |  |  |  |  |
| If I do not know what is in a particular food, I will not taste it |  |  |  |  |  |  |
| I like foods from different cultures |  |  |  |  |  |  |
| Ethnic foods seem too strange to me to eat |  |  |  |  |  |  |
| At events that include meals, I will taste new dishes |  |  |  |  |  |  |
| I am afraid to eat things I have not eaten before |  |  |  |  |  |  |
| I am very picky about the food I eat |  |  |  |  |  |  |
| I eat almost everything |  |  |  |  |  |  |
| I would love to try new ethnic restaurants |  |  |  |  |  |  |

|  | )(1)Strongly disagree | (2) | (3) | (4) | (5) | (6) Strongly agree |
| --- | --- | --- | --- | --- | --- | --- |
| I do not feel comfortable when I find myself in new situations |  |  |  |  |  |  |
| When I'm out of the house, I want to return home to my familiar environment |  |  |  |  |  |  |
| I am afraid of the unknown |  |  |  |  |  |  |
| I feel very uncomfortable in new situations |  |  |  |  |  |  |
| When I'm on vacation, I can not wait to get home already |  |  |  |  |  |  |
| I avoid talking to people I do not know |  |  |  |  |  |  |
| I feel uncomfortable when I am in an environment unfamiliar to me |  |  |  |  |  |  |
| I do not like to sit next to someone I do not know |  |  |  |  |  |  |

Sometimes, we do not behave according to our routine (unusual periods, special days). In the following questions, you will be asked about your lifestyle in the last week. Please answer as accurately as possible, even if this week does not represent your lifestyle in general. There are no right or wrong answers. The information collected will remain private and will be used only for this study.

**This sectionk repeated all questions from 12-27 included; the only difference is that we asked specifically regarding **last week.**

1. All the questionnaires were administered using a computer [↑](#footnote-ref-1)
