# Supplementary material for: Promoting Healthy Eating Behaviors by Incentivizing Exploration of Healthy Alternatives
Source: Front Nutr. 2021 Jun 15;8:658793. doi: 10.3389/fnut.2021.658793 (PMC8239191; doi:10.3389/fnut.2021.658793)
Supplement: Supplementary file 2 [file Table_2.DOCX]

**Supplemental material 2: Additional questions that followed the post-intervention questionnaire:**

We would like to learn from your experience if we will want to run a similar experiment in the future

Please answer the following statements

|  | (1) Not clear at all | (2) | (3) | (4) | (5) | (6) Entirely clear |
| --- | --- | --- | --- | --- | --- | --- |
| (1) Definition of the experimental requirements |  |  |  |  |  |  |
| (2) Definition of what is considered as a salad |  |  |  |  |  |  |

How easy/ difficult were the following:

|  | (1) Very easy | (2) | (3) | (4) | (5) | (6) Very difficult |
| --- | --- | --- | --- | --- | --- | --- |
| 1. Finding salads |  |  |  |  |  |  |
| 1. Preparing salads |  |  |  |  |  |  |
| 1. Meet the requirements of the experiment |  |  |  |  |  |  |

Please rate the following sentences: from 1 not at all to 6 very much

|  | 1. Not at all | (2) | (3) | (4) | (5) | (6) Very much |
| --- | --- | --- | --- | --- | --- | --- |
| (1) Would you like to participate in a similar experiment again? |  |  |  |  |  |  |
| (2) Would you recommend to a friend to participate in such an experiment? |  |  |  |  |  |  |
| (3) How much effort did you put into the experiment |  |  |  |  |  |  |
| (4) How much did you enjoyed the salads you ate |  |  |  |  |  |  |
| 5) Do you think the experiment helped you eat healthier |  |  |  |  |  |  |
| (6) How much did you enjoy the course of the experiment |  |  |  |  |  |  |

Rate your exposure to new foods during the experiment

|  | (1) Not at all | (2) | (3) | (4) | (5) | (6) All the time |
| --- | --- | --- | --- | --- | --- | --- |
| 1. Were you exposed to new foods during the experiment |  |  |  |  |  |  |

Please indicate:

|  | 1. Payment too low | (2) | (3) | (4) | (5) | (6) Payment too high |
| --- | --- | --- | --- | --- | --- | --- |
| The extent to which the payment for participating in the experiment is commensurate with the required investment |  |  |  |  |  |  |

On average, how much time (in minutes) did you invest each day in the experiment? _____

Please note that the following question and your answer to it will not affect your payment in any way, but it is important to us that you will answer honestly!

|  | (1) Not at all | (2) | (3) | (4) | (5) | (6) All the time |
| --- | --- | --- | --- | --- | --- | --- |
| Did you cheat during the experiment |  |  |  |  |  |  |

If you state that you cheated please explain how: _________________

Please write any recommendation or comments that you think should be considered if we run a similar experiment again _______________
