# Supplementary material for: Promoting Healthy Eating Behaviors by Incentivizing Exploration of Healthy Alternatives
Source: Front Nutr. 2021 Jun 15;8:658793. doi: 10.3389/fnut.2021.658793 (PMC8239191; doi:10.3389/fnut.2021.658793)
Supplement: Supplementary file 3 [file Table_3.docx]

**Supplemental material 3: One year follow-up questionnaire**

Thank you for your willingness to participate.

Participant ID

Height

Weight

Last week, how many times on average did you eat (drink):

|  | (1)  Less than once a day | (2)  Once a day | (3)  2  times a day | (4)  3 times a day | (5)  4-5 times a day | (6)  Almost once a day | (7)  Every day | (8)  More than once a day |
| --- | --- | --- | --- | --- | --- | --- | --- | --- |
| "junk food" (such as pizza, hamburger, shawarma) |  |  |  |  |  |  |  |  |
| Sweets and snacks (such as cakes, cookies, pastries) |  |  |  |  |  |  |  |  |
| Healthy snacks (such as oatmeal, unsweetened nuts) |  |  |  |  |  |  |  |  |
| Fresh fruits (such as apples, bananas, oranges) |  |  |  |  |  |  |  |  |
| Fresh vegetables not as a part of a salad (such as pepper, cucumber, tomato) |  |  |  |  |  |  |  |  |
| salads (a mixture that includes at least two fresh vegetables and / or fresh leaves) |  |  |  |  |  |  |  |  |
| Alcohol |  |  |  |  |  |  |  |  |
